# Security for the Masseur and the Masseuse

**Published:** 1921-04-23

**Authors:** 


### Security for the Masseur and the Masseuse.

The Chartered Society of Massage and Medical Gymnastics has an admirable year's work to its credit, as reported on page 72: The only note of disappointment lies in the failure of the great bulk of the certificate-holders from the Incorporated Society of Trained Masseuses to register; the numbers show a total of 2,148 registrations only, out of a possible 6,735. Of these a proportion will, no doubt, come forward before July, in order to secure that their names may appear in the first printed Register, but, even allowing for those who have dropped out of practice, the figures show a strange lack of the self-protective instinct which should impel workers to take advantage to the utmost of the privileges won for them. A somewhat similar lack of corporate fellowship was brought to light by Mr. Louis H. M. Dick when speaking of the value of the small Benevolent Fund available for members and the need for increasing its resources. One farthing a week contributed by all members would render it able to cope with all cases of distress likely to arise. Lack of forethought and lack of imagination are responsible both for the neglect of registration now it is within reach and neglect of the ample facilities for insurance—life, accident, and illness—proffered by the Chartered Society in the arrangements it has made.

